# The Mediating Role of Orthorexia in the Relationship between Physical Activity and Fear of COVID-19 among University Students in Poland

**DOI:** 10.3390/jcm10215061

**Published:** 2021-10-29

**Authors:** Cezary Kuśnierz, Aleksandra Maria Rogowska, Aleksandra Kwaśnicka, Dominika Ochnik

**Affiliations:** 1 Faculty of Physical Education and Physiotherapy, Opole University of Technology, 45-758 Opole, Poland; c.kusnierz@po.edu.pl; 2 Institute of Psychology, University of Opole, 45-052 Opole, Poland; aleksandra.kwasnicka@uni.opole.pl; 3 Faculty of Medicine, University of Technology, 40-555 Katowice, Poland; dominika.ochnik@wst.pl

**Keywords:** fear of COVID-19, orthorexia nervosa, physical activity, university students, healthy lifestyle

## Abstract

Previous research showed that the COVID-19 pandemic has a significant impact on the wellbeing and lifestyle of populations worldwide, including eating and physical activity (PA) patterns. The present study aims to examine the mediating effect of orthorexia on the relationship between PA and fear of COVID-19. A sample of 473 university students from Poland of a mean age of 22 years (*M* = 22.04, *SD* = 2.90, 47% of women) participated in the cross-sectional online survey study. Continuous variables were measured using the Fear of COVID-19 Scale (FCV-19S) and the Test of Orthorexia Nervosa (TON-17), while categorical variables divided participants into the physically active and inactive group regarding WHO criteria (150 min per week). Weak gender differences were found. Active people showed lower fear of COVID-19 and higher orthorexia scores than those inactive. Orthorexia was found as a suppressor variable, which increases the negative predictive value of PA on fear of COVID-19. The model of cooperative suppression explained 7% of FCV-19S. The mechanism of mediation showed that health-related behavior could help reduce fear of COVID-19, but caution is necessary for people with addictive behavior tendencies. Universities should support university students by offering programs focused on increasing healthy lifestyles and improving wellbeing.

## 1. Introduction

### 1.1. Impact of COVID-19 on Lifestyle

The new coronavirus disease “COVID-19” was identified in December 2019 in the Wuhan region of China and has since spread worldwide in spring 2020. In Poland, the Coronavirus pandemic 2019 (COVID-19) began on 4 March 2020 [1]. Lockdown-related social distancing and numerous restrictions disrupted everyday life, including social and family life, school, or work. A systematic review and meta-analysis [2] found that the pooled prevalence of anxiety equals 33%, while for depression it is 28% in the public, with such risk factors as female gender, nurses, lower socioeconomic status, high risks of contracting COVID-19, and social isolation. Muyor-Rodríguez et al. [3] suggested that college students are considered an especially vulnerable group for mental health problems during the COVID-19 pandemic. Indeed, a nationwide study indicates that at least one mental health problem (including high stress, anxiety, or depression) was found in 45% of Chinese college students [4] and 42.8% of university students from France [5]. Furthermore, high levels of stress, anxiety, and depression were found in university students from many countries [6,7,8]. In addition, a systematic review and meta-analysis showed that mental health problems in university student populations increased during the COVID-19 pandemic period compared to pre-pandemic times [9].

The COVID-19 pandemic significantly impacted lifestyle, including changes in dietary habits and physical activity (PA) patterns [10,11,12,13,14,15,16,17,18,19,20]. Izzo et al. [17] found changes in eating habits among 33.5% of respondents (out of which 81% reported an increase in frozen food consumption) and physical activity reduction in 70.5% of the Italian population during the COVID-19 lockdown. A scoping review [11] found an increase in unfavorable dietary habits (e.g., increased alcohol, sweets, fried food, snacks, processed foods consumption, and reduced fresh produce intake), weight gain, and a reduction in physical exercise during the pandemic. Cecchetto et al. [12] showed that isolation and lockdown have adverse effects on eating behavior and emotional wellbeing through increased emotional distress, binge eating, and higher BMI scores. The impact of the coronavirus pandemic on eating and exercise behaviors was also investigated in an Australian sample among individuals with an eating disorder and the general population [19]. Increased restricting, binge eating, and purging were found in both groups, while a higher level of exercise behavior was reported in people with eating disorders (EDs) than the general population. The present study aims to examine the relationship of physical activity with orthorexia (eating disorder relying on restrictive eating tendencies) and fear of COVID-19 among a sample of university students from Poland.

### 1.2. Healthy Lifestyle among University Students

Lockdown and corresponding social isolation also affected the dietary patterns of university students, which can lead to a higher frequency of excessive weight and obesity [13,21,22]. However, increased level of physical activity during lockdown was usually related to a healthier diet among university students [13]. The level of PA decreased significantly during the COVID-19 pandemic [5]. In the sample of Turkish students, 38% were physically active and met the WHO criterion (over 150 min per week) before the pandemic, while only 13% remained active [5]. Prevalence of low PA level was 37.1% among Swiss university students, and 36.1% reported prolonged sitting time (>8 h/day) during the COVID-19 lockdown [23]. Among university students from Ukraine, 43% were engaged in PA ≥ 150 min weekly, 24% met the criteria of General Anxiety Disorder (GAD), while 32% met the criteria for depression during the first wave of the COVID-19 pandemic [24]. Furthermore, students reported being more involved in PA before the COVID-19 outbreak than during the lockdown.

Studies show that university students do not exhibit satisfactory health-oriented behaviors in terms of their physical activity, diet, alcohol and drug use, preventive practices, mental health, stress, and social relationships. However, female university students performed higher in health-related behavior than men [25,26,27]. A recent study examined the health-related behaviors of university students from Poland, Croatia, Turkey, Lebanon, Spain, Romania, and Italy [28]. Self-rated health was positively related to the female gender, daily breakfast eating, physical activity, and time spent studying, while negatively with BMI, stress, and smoking.

The study indicates that 68.4% of men and 48.4% of women reported practicing physical activity in a representative sample of students from a Spanish university [29]. Physical activity was positively associated with consuming more fruits. Romaguera et al. [29] found that physically active students tended to engage in other healthy habits. In addition, a more recent study indicated that most university students from Spain (69.6%) were involved in physical activity, which was related to less sedentary behavior [30]. Badicu [31] found the prevalence of low, moderate, and high levels of PA in 17.65%, 30.58%, and 51.76% of men, and also in 23.02%, 43.16%, and 33.81% of women, respectively, in a sample of students of the Faculty of Physical Education and Sport. Many students demonstrated a good sleep quality and a high level of PA. However, some differences were presented depending on the year of study, gender, and academic field. A higher percentage of men than women was engaged in a high PA level. A medium correlation was presented between PA level and sleep quality. Dąbrowska et al. [32] showed that 46% of Polish physiotherapy students had a high PA and 54% a moderate PA, while a low level of physical activity was shown in 26% of medical students.

### 1.3. Orthorexia Nervosa

Orthorexia Nervosa (ON) can be defined as “a pathological obsession, fixation or preoccupation with healthy food” [33] (p. 1); however, researchers have discussed different diagnostic criteria [33,34,35,36,37]. As a trait, ON is characterized by restrictive and avoidant eating behavior and a tendency to pathological obsession and preoccupation with healthy, strictly organic, and biologically pure foods [36]. The ON is not yet included in the International Statistical Classification of Diseases and Related Health Problems (ICD-10) or the Diagnostic and Statistical Manual of Mental Disorders (DSM-5), because it is not clear whether ON is as a single syndrome of an eating disorder (EDs) or a variant of avoidant/restrictive food intake disorder (AFRID), anorexia nervosa (AN), obsessive-compulsive disorder (OCD), somatic symptom disorder, obsessive-compulsive personality disorder (OCPD), psychotic spectrum disorders, or general anxiety disorder [38,39,40,41,42].

The prevalence of orthorexia depends on the diagnostic cut-off criteria, the instrument used, or geographic region, and ranges between 1% to 89%, as suggested by review studies [43,44,45]. However, Varga et al. [45] found the average prevalence of ON to be 6.9% in the general population, while rates between 1% and 7% were estimated by using Düseldorf Orthorexia Scale (DOS ≥ 30) [46], and 5.5% using Test of Orthorexia Nervosa (TON-17 ≥ 61) [47] in the Polish sample. However, the prevalence of ON was rarely assessed among university students. We have found only two such studies. Brytek-Matera et al. [48] found ON prevalence rates of 2.3% and 2.9% (using DOS) among university students’ Spanish and Polish samples. Orthorexic tendencies were present in 65.31% of 320 university students from the Lebanon (ORTO-15 < 40) [49]. Reynolds [50] found the prevalence rate of 6.5% of Australian adults at a Sydney university, using the cut-off score of ORTO-15 < 35 and diagnostic criteria. Comparing two measurement methods ORTO-11-ES and DOS-ES, Parra-Fernández et al. [51] showed that the prevalence of ON was 25.2% or 10.5%, respectively, among Spanish university students.

Research indicates that ON may lead to poor physical and mental health. In particular, ON is positively related to eating disorders (EDs), obsessive-compulsive traits, stress, anxiety, and depressive symptoms, and negatively associated with psychological wellbeing and life satisfaction [40,52,53]. Recent studies also found correlations between ON and EDs, healthy behaviors, anxiety, obsessive-compulsive disorder (OCD), and depression [47,54].

### 1.4. Fear of COVID-19

One of the most common emotions emerging during the pandemic is the fear of COVID-19, which can include contracting the disease or infecting loved ones, death of loved ones, severe course of illness in loved ones, healthcare failure, and the consequences of the pandemic at an individual and social level [55]. Tzur Bitan et al. [56] found two dimensions of fear of COVID-19 using the Fear of COVID-19 scale (FCV-19S), namely emotional fear reactions and symptomatic expressions of fear. Fear of COVID-19 is positively correlated with stress, depression, anxiety (considered a trait and a state), chronic illness, germ aversion, being in an at-risk group, and having a family member die of COVID-19, while it is negatively related to life satisfaction [2,56,57,58].

Fear of COVID was examined in university students [2,59,60,61,62,63,64,65]. Norwegian nursing students showed higher levels of fear of COVID compared to the general reference population [61]. Fear of COVID-19 (measured by FCV-19S) was positively related to psychological distress and negatively associated with general health and quality of life (QoL). Extremely high fear of COVID-19 can lead to coronaphobia. Arora et al. [66] defined coronaphobia as “an excessive triggered response of fear of contracting the virus causing COVID-19, leading to accompanied excessive concern over physiological symptoms, significant stress about personal and occupational loss, increased reassurance and safety-seeking behaviors, and avoidance of public places and situations, causing marked impairment in daily life functioning. The triggers involve situations or people involving probability of virus contraction, such as meeting people, leaving the house, traveling, reading the updates or news, falling ill, or going for work outside” (p. 2). Coronaphobia consists of three components: (1) physiological response of fear, which can appear with such symptoms as palpitations, tremors, difficulty in breathing, dizziness, change in appetite, and sleep; (2) cognitive response and preoccupation with threat-provoking cognitions, which can also trigger emotional response (e.g., sadness, guilt, anger); (3) behavioral response by engagement in avoidance behaviors (e.g., avoiding gatherings, shopping, public places, and social situations) or excessive engagement in health-related behaviors (e.g., washing hands, preparing healthy nutrition).

### 1.5. The Relationships between Physical Activity, Orthorexia, and Fear of COVID-19

Rodgers et al. [67] assumed fear of COVID-19 might increase the risk of EDs. People can pursue restrictive diets focused on increasing immunity. Increased stress and negative emotions due to the pandemic and social isolation can also increase the risk of ED. Evaluating and assessing these factors in different cultural environments is crucial to better understand the impact of the pandemic on risk and recovery in EDs. Indeed, research indicates fear of COVID-19 was related to greater bulimic behavior among Italian college students [68]. A high prevalence of orthorexia (67% in men and 83.2% in women, using ORTO-11) and anxiety symptoms (62.4% in men and 95.4% in women, using GAD-7) was found among adults during the COVID-19 pandemic [69]. Moreover, orthorexia and anxiety symptoms were positively associated. We assume that orthorexic behavior focused on a healthy diet can be a coping strategy to reduce fear of COVID-19.

Numerous studies showed a positive association between ON and physical activity or exercise addiction [49,70,71,72,73,74,75,76,77,78,79,80,81,82,83,84,85]. Segura-García et al. [81] indicated athletes presented higher ON scores than the control group. In addition, professional sport involvement was a predictor of ON [81]. ON was also predicted by frequent exercising in a sample of Portuguese fitness participants [70] and by endurance sport practice (sports with predominantly aerobic activity > 150 min/week) in Italian athletes [71]. A recent systematic review and meta-analysis [82] showed a small correlation between ON and exercise and a medium correlation between ON and exercise addiction. Strahler et al. [82] suggest that comorbidity between ON and exercise addiction should be explained in future research, focusing on clinical relevance, underlying mechanisms, vulnerability, and risk factors.

### 1.6. The Current Study

The present study aims to examine the mediating role of orthorexia on the relationships between PA and fear of COVID-19. Previous research found that PA is negatively associated with anxiety and depression as well as negative emotions in university students during the COVID-19 pandemic [6,24,86,87]. The physically inactive group had higher scores of anxiety and depression than the physically active group [24]. Moreover, insufficient physical activity was a predictor of high anxiety among university students from Poland and Ukraine during the COVID-19 pandemic [6]. The total physical activity level and low-intensity physical activity were inversely associated with depressive symptoms in Chinese college students [86]. A longitudinal study of college students in China indicates that physical activity directly alleviated general negative emotions [87]. We assume there are inverse relationships between PA and fear of COVID-19. However, extreme PA level or exercise addiction may be positively related to fear of COVID-19. A healthy diet and exercising are two correlated dimensions of a healthy lifestyle. Orthorexia can mediate the relationship between PA and fear of COVID-19 since being involved in both orthorexia and compulsive exercise can be used as a coping strategy to decrease general anxiety and fear of COVID-19. Due to the COVID-19 pandemic-related restrictions in social contacts, the paper and pencil versions of the questionnaires are impossible to complete. Therefore, all variables of interest (including PA, orthorexia, and fear of COVID-19) will be measured using an online survey with standardized questionnaires developed or validated in the Polish cultural context.

## 2. Materials and Methods

### 2.1. Study Design

An online questionnaire was used for a cross-sectional self-report survey. The invitation to participate in the study was posted on the university e-learning website from 14 April to 16 June. As e-learning was the main software for attending online classes during the lockdown, the invitation was aimed at the university’s entire student population. Students were asked to participate in the study by anonymously completing a questionnaire. Participants’ personal data was not obtained, and no compensation was offered as an incentive to participate. The sample size was determined a priori using G*Power ver. version 3.1.9.4. for Windows, Uiversität Kiel, Germany [88]. In order to detect a medium effect size ϕ = 0.30 with given 95% power (1-β error probability) in a bivariate correlation (two tails), α = 0.05, G*Power suggests 138 participants are needed in the study group (critical *r CI* = –0.167, 0.167; power = 0.950). When Student’s *t*-test (two tails) was considered for two independent gender samples (women and men), with a moderate effect size *d* = 0.50, 95% power (1-β error probability) and α = 0.05, the required sample size should consist of 210 people (non-centrality parameter δ = 3.623; critical *t*(208)= 1.971, minimal gender sample size *n* = 105, total sample size *N* = 210, power = 0.950). For regression analysis, an expected sample size is 107, if considering two predictors, a medium effect size *f*^2^ = 0.15, with given 95% power (1-β error probability) in a bivariate correlation (two tails), and α = 0.05, with critical *F*(2, 104) = 3.08, and non-centrality parameter λ = 16.05.

### 2.2. Participants

All Opole University of Technology students (about 6000 people) willing to participate and above 18 years old were able to participate in the study. The total number of responses to the invitation was 490, completed surveys was 482, while eight people refused to participate in the study at the informed consent stage (1.6% of refusal rate). However, to minimize the source of bias and allow gender comparisons, only students who disclosed their gender as either male or female were included in the final sample, which consisted of *N* = 473 participants, out of which *n* = 222 (47%) were female and *n* = 251 (53%) were male. Participants’ age ranged between 19 and 47 (*M* = 22.04, *SD* = 2.90). Students were members of one of six departments: Civil Engineering and Architecture, Economics and Management, Electrical Engineering, Automatic Control and Informatics, Production Engineering and Logistics, Mechanical, and Physical Education and Physiotherapy. Details are shown in Table 1.

**Figure 1:**
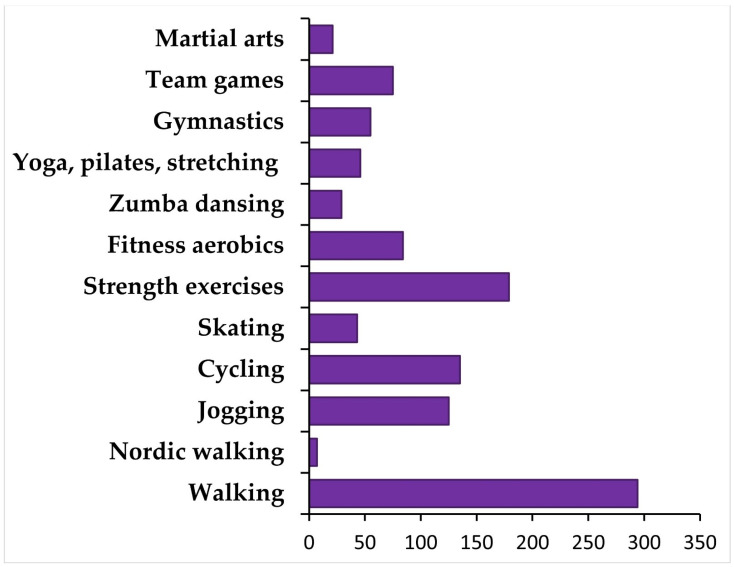
Number of university students engaging in different physical activities (*N* = 473).

Most university students reported engagement in walking (*n* = 294, 62.16% of the total sample), strength exercises (*n* = 179, 37%), cycling (*n* = 135, 28%), and jogging (*n* = 125, 26.42%). Selected forms of PA are shown in Figure 1. Among participants, 7.4% (*n* = 35) did not undertake any physical activity during the last week, 13.5% (*n* = 64) were involved in PA on one day, 19.2% (*n* = 91) on two days, 20.5% (*n* = 97) three days, 19.2% (*n* = 91) four days, 9.7% (*n* = 46) five days, 4.9% (*n* = 23) six days, and 5.5% (*n* = 26) seven days a week. In the total sample (*N* = 473), university students were engaged in PA on average 3 days a week (*M* = 3.07, *SD* = 1.82, ranging from 0 to 7 days in the last week). The mean duration of PA during one typical session was approximately one hour (*M* = 57.51, *SD* = 44.68, ranging from 0 to 420 min daily), whereas 200 min weekly (*M* = 201.29, *SD* = 225.78, ranging from 0 to 1800 min weekly). A sample of 222 students were identified as those who exercised a minimum of 150 min per week (Active group, 46.9% of the total sample), and 251 did not meet the criterion of a minimum of 150 min of PA per week (Inactive group, 53%).

### 2.3. Measures

The Fear of COVID-19 Scale (FCV-19S) [57] is a seven-item scale designed to measure the extent to which people are afraid of negative outcomes of the global COVID-19 pandemic. It consists of statements concerning different symptoms of fear caused by the COVID-19 virus. Participants assess how strongly they agree with said statements on a scale from 1 (*strongly disagree*) to 5 (*strongly agree*), and the final score is calculated by adding up each score. The internal consistency of the FCV-19S was good in the original study (Cronbach’s α = 0.82) [57]. The reliability coefficient was even better in the present sample (Cronbach’s α = 0.88).

Orthorexia was measured with a 17-item Test of Orthorexia Nervosa (TON-17) questionnaire, with a response scale ranging from 1 (*totally disagree*) to 5 (*totally agree*) [47]. All items form a general score (by addition) and can also be divided into three subscales: control of food quality (CFQ), fixation on health and healthy lifestyle (FHHL), and disorder symptoms (DS). A cut-off score TON-17 ≥ 61 is used to classify orthorexia [47]. The reliability coefficient in the previous study was 0.82, 0.79, 0.80, and 0.81, for CFQ, FHHL, DS, and of the total TON-17, respectively [47]. In the present study, the reliability coefficient ranged between 0.73 to 0.81 (Cronbach’s α = 0.73 for CFQ, α = 0.73 for FHHL, α = 0.78 for DS, and α = 0.81 for the total score of TON-17).

Physical Activity (PA) was assessed by two questions concerning the frequency and duration of physical activities in the past week. The responses were then multiplied to form the number of active minutes for the last week. The WHO global recommendation of PA maintaining health among adults between 18 and 64 years old is to do at least 150 min of moderate-intensity aerobic PA or at least 75 min of vigorous-intensity aerobic physical activity throughout the week, or an equivalent combination of moderate- and vigorous-intensity activity [89]. However, it is difficult to assess the differences between low, moderate, and vigorous-intensity PA for an average non-expert person. Moreover, involvement in PA should be tailored to individual preferences, capabilities, opportunities, and circumstances and should be appropriate for people with chronic diseases or physical disabilities. Therefore, we abandoned the question regarding intensity and assumed that the active group would include those students who exercise regularly at least 150 min per week, regardless of the degree of intensity. This approach was used in the previous study [24]. Active participants (PA ≥ 150 min a week) were coded as 0, and Inactive (PA < 150 min a week) were coded as 1.

### 2.4. Statistical Analyses

Firstly, the reliability analysis of utilized scales was performed. All scales and subscales showed at least good reliability [90] as measured by Cronbach’s α. Descriptive statistics, such as range of scores, mean (*M*), median (*Mdn*), standard deviation (*SD*), skewness, and kurtosis, were computed for all scales, along with Shapiro–Wilk’s normality test. Most variables showed marginal deviations from the normal curve, the exceptions being FCV-19S and disorder symptoms scale of the TON-17, which were strongly positively skewed and leptokurtic. However, since the sample size was large and a value ±2 is acceptable [91] (p. 114), parametric tests were used in further statistical analysis. As the study’s main goal was to test the mediation effect using Hayes’ PROCESS macro, based on bootstrapping technics (which is a nonparametric analysis), the assumption of normality distributions is not required.

An association between gender and PA (considered as categorical variables) was examined using a contingency table and Pearson’s χ^2^ test, with ϕ coefficient to test effect size. The Student’s *t*-test was used to examine differences between genders and active versus nonactive participants, with Cohen’s *d* to examine effect size. Interaction of gender and physical activity was checked using two-way ANOVA, and Pearson’s *r* was calculated to assess the associations between variables. The mediating role of orthorexia in the relationship between physical activity and fear of COVID was tested using Model 4 of PROCESS v. 3.5. Macro for SPSS, designed by Hayes [92]. A bootstrapping procedure with 5000 resampling was used to assign measures of accuracy to sample estimates. All analyses were performed using Statistical Package for the Social Sciences (IBM SPSS Statistics, ver. 25, 2019, Predictive Solutions Sp. z o.o., Kraków, Poland). The sample size exceeded the needed number of participants, which increased the power to 1.00 for Student’s *t*-test, Pearson’s correlation, and regression analysis, as suggested by G*Power posthoc test.

## 3. Results

### 3.1. Descriptive Statistics

In the first step of statistical analyses, descriptive statistics were computed, and distributions of variables were examined. Values of skewness and kurtosis exceeded the absolute value of +1 for two of the variables but did not exceed ±2, as shown in Table 2. The prevalence of orthorexia was determined by using a cut-off score of TON ≥ 61. In the total sample, 21 students identified with a score of 61 or more, which is 4.44% of ON prevalence. Among participants, 222 people met the minimum 150 min PA per week (47% of the total sample), while 251 students (53%) were included in the physically inactive group.

### 3.2. Group Comparisons

A contingency table was created to compare frequencies of physically active men (*n* = 128) and women (*n* = 94), with inactive male (*n* = 123) and female (*n* = 128) participants. The Person’s χ^2^ test (two-tailed) did not show significant associations between PA and gender, χ^2^(1) = 3.54, *p* = 0.060, ϕ = 0.09. Gender differences were examined in regards to all measured dimensions (Table 3). Women showed significantly higher levels of fear of COVID and fixation on health and healthy diet (which is one of the subscales of orthorexia) than men. Men scored higher than women in disorder symptoms of orthorexia. However, all differences were presented with a small effect size.

**Figure 2:**
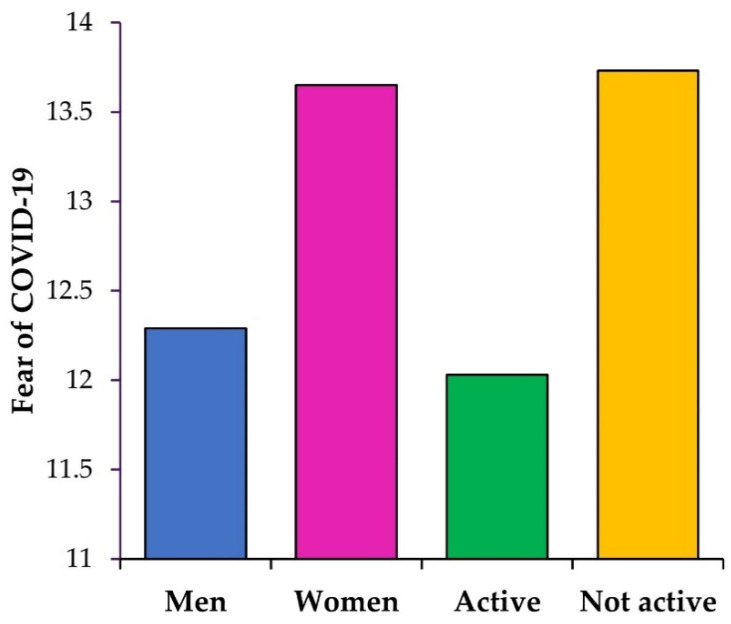
A comparison of mean scores in fear of COVID-19 among women, men, and active and inactive university students.

Fear of COVID also showed significant differences depending on the activity level (see Table 3 and Figure 2 for more details). Inactive participants scored higher in fear of COVID-19 than those exercising at least 150 min a week. For orthorexia and its two subscales (control of food quality and fixation on health and healthy diet), the opposite effect was found—active people obtained higher scores of orthorexia than those who were inactive. A medium effect size was found for FCV-19S and FHHL, while a small effect was found for a total score of the TON-17 and CFQ subscale.

Interaction between those two dichotomous variables was checked, but the two-way ANOVA did not show a significant interaction effect of gender and PA on Fear of COVID-19, *F*(1, 469) = 0.01, *p* = 0.917; orthorexia (total score of the TON-17), *F*(1, 469) = 0.06, *p* = 0.800; control of food quality *F*(1, 469) = 0.34, *p* = 0.563; fixation on health and healthy diet *F*(1, 469) = 0.92, *p* = 0.338; and disorder symptoms *F*(1, 469) = 3.53, *p* = 0.061.

### 3.3. The Relationships between PA, Orthorexia, and Fear of COVID-19

Fear of COVID is significantly correlated with orthorexia (*r* = 0.20, *p* < 0.001) and its two subscales, control of food quality (*r* = 0.16, *p* < 0.001) and disorder symptoms (*r* = 0.19, *p* < 0.001). All correlations are positive, suggesting that a higher risk of orthorexia is connected with a higher level of fear of COVID. However, the strength of these associations is weak.

**Figure 3:**
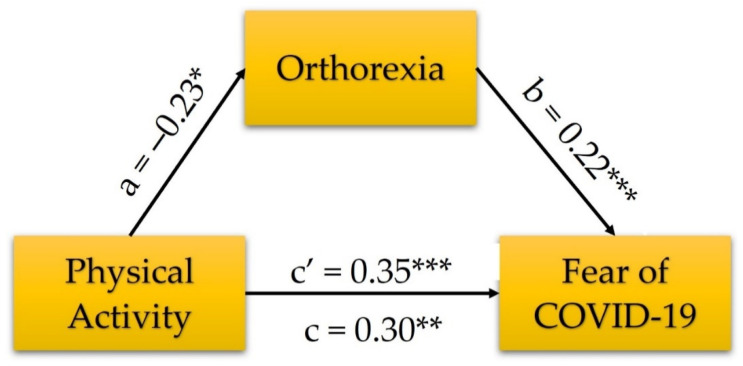
Orthorexia as a mediator of the relationship between physical activity and fear of COVID with standardized coefficients. * *p* < 0.05, ** *p* < 0.01, *** *p* < 0.001.

As the final step of analyses, the mediating effect of orthorexia on the relationship between PA and fear of COVID-19 was verified (Figure 3). As it was assumed, physically inactive participants scored higher in fear of COVID, *b* = 1.70, *SE b* = 0.52, β = 0.30, *t* = 3.29, *p* < 0.001, *R*^2^ = 0.02, *F*(1, 471) = 10.81 (total effect). Higher orthorexia scores can be predicted in physically active people, *b* = −2.24, *SE b* = 0.89, β = 0.23, *t* = −2.53, *p* = 0.012, *R*^2^ = 0.01, *F*(1, 471) = 6.39. When orthorexia was included in the regression model, all variables were significantly and positively associated with fear of COVID-19, increasing variance explained to 7%, *R*^2^ = 0.07, *F*(2, 470) = 17.49. Orthorexia was found as a positive predictor of fear of COVID-19, *b* = 0.13, *SE b* = 0.03, β = 0.22, *t* = 4.86, *p* < 0.001, Boot *M* = 0.13, Boot *SE* = 0.03, 95% *CI* = (0.064; 0.186). Moreover, physical inactivity was a predictor of higher fear of COVID-19, *b* = 1.98, *SE b* = 0.51, β = 0.35, *t* = 3.91, *p* < 0.001, Boot *M* = 1.97, Boot SE = 0.51, 95% CI = 0.987; 2.994 (direct effect). Bootstrap estimate for indirect effect was −0.28, Boot *SE* = −0.13, 95% *CI* = −0.553; −0.059. Since the Boot *CI*s do not include 0, we conclude that all associations are significant, and mediation is confirmed. However, the regression coefficient value increased when orthorexia was included in the regression model, which indicates cooperative suppression [93] or reciprocal suppression [94].

## 4. Discussion

### 4.1. Associations between Variables

This study aimed to examine the mediating effect of orthorexia on the relationships between PA and fear of COVID-19. This study found evidence of a significant reciprocal (cooperative) suppression effect in regression analysis. Orthorexia is a suppressor variable that increases the predictive value of PA on fear of COVID-19 by its inclusion in a regression equation [95]. Overall, a high level of PA contributes to better wellbeing. In contrast, orthorexia is related to the worst mental and physical health. However, high orthorexia is associated with a high PA level. Therefore, orthorexia demonstrates a suppressing effect on the relationship between PA and fear of COVID-19. Because people with high orthorexia are more likely to have a high fear of COVID-19, orthorexia as a mediator suppresses some parts of PA variance (most likely those related to excessive PA) and increases PA’s effect on fear of COVID. Orthorexia as a suppressor contributed to an additional 5% of the variance beyond the separate direct effect of PA on fear of COVID-19.

These results establish that the PA contains three distinct, opposing components: low PA level is unhealthy, moderate PA level is beneficial for health, and exercise addiction has adverse consequences for mental and physical health. This complexity of antagonistic tendencies in PA cannot be taken into account in a simple correlation. The present findings allowed us to distinguish these opposing processes exclusively during the COVID-19 crisis.

Although physical inactivity predicts high fear of COVID-19, extremely high PA (indicating exercise addictive tendencies) can also increase fear of COVID-19, likely because (together with orthorexia) it includes a pathological anxiety component. On the other hand, healthy compulsive behavior related to diet (orthorexia) and PA (exercise addiction, EA) may be performed to reduce fear of COVID-19, and a vicious circle can start to spin. Increasing orthorexic and EA behaviors decreases the fear of COVID-19 for a moment, but continuous concerns about health trigger a further reaction in a more compulsive way. Solymosi et al. [96] distinguish functional and dysfunctional fear of COVID-19. Functional concerns motivate a behavioral response that helps people manage any insecurity to unharm their quality of life. In contrast, dysfunctional fear can be presented when the quality of life is subjectively reduced either by worry or precautionary behavior (or both). Further research is necessary to replicate the present research. Additionally, a separate analysis could be conducted in the future in distinct groups: inactive people, those with moderate PA levels, and persons with exercise addiction.

Numerous studies showed an association between excessive exercise and ON. Among people with orthorexic behaviors, 5.2% showed a sedentary lifestyle, 45% were lightly physically active, 41.15% were active, while 8.6% were very active [49]. For comparison, 9.9% demonstrated a sedentary lifestyle among students with regular eating behavior, 46.85% light physical activity, 36.93% were active, and 6.3% very active physically. Clifford and Blyth [72] found higher ON prevalence among athletes who undertake high volumes of exercise. PA (measured by using IPAQ-SF) was significantly associated with ON tendencies (ORTO-15) among Dutch university students [74]. When study majors were compared, a higher proportion of people with ON were found in exercise science students (in particular among men) than in business students [78]. Furthermore, Oberle et al. [79] indicated that university students who scored high in orthorexia (using EHQ) were internally driven to exercise to improve their physical and mental health. Orthorexia was positively correlated with aerobic and strength-training exercise levels, exercise addiction, internal exercise motivation, and exercise motivation for psychological, social, health, and body improvement.

Kiss-Leizer et al. [77] suggested that obsessive features of sports activities play an essential role in ON. Indeed, Rudolph [80] found a significant positive correlation between ON (using DOS) and exercise addiction among German members of fitness studios. Research indicates that social desirability, guilt over skipping training, and health anxiety were the strongest predictors of ON. Almeida et al. [70] showed that ON is associated with other non-dietary behaviors focused on a healthy lifestyle and aesthetic concerns, such as physical appearance and frequent exercising. In particular, frequent exercising was a predictor of ON in the sample of Portuguese fitness participants. Bert et al. [71] found that ON (using EHQ) can be predicted by endurance sport practice (sports with predominantly aerobic activity > 150 min/week) in Italian athletes (*b* = 2.407, 95% *CI* = 0.27;4.54). Moreover, Yılmaz et al. [84] found higher orthorexic tendencies (ORTO-15) in participants who regularly performed physical exercises than those diagnosed with obsessive-compulsive disorder (OCD) and healthy individuals who did not perform physical exercises.

### 4.2. Orthorexia, Fear of COVID-19, and PA among University Students

We found the prevalence of orthorexia in 4.44% of university students, while criteria of a minimum of 150 min PA per week were reported in 47% of the sample. The prevalence of orthorexia among university students is within the range of 1–7%, which was found in some review studies [45,46], and is lower than in the previous study, which used TON-17 (5.5% in the general population) [47], but slightly higher than among Polish and Spanish students by using DOS (2.9% and 2.3%, respectively) [48]. The prevalence of ON was previously discussed as challenging to compare with other studies since various measurement tools have different cut-off criteria. Cross-cultural differences may also be important for it [43,44,45]. Di Renzo et al. [14] examined the impact of the early stage of the COVID-19 pandemic on eating habits and lifestyle changes among the Italian population. The research found that 15% of participants turned to farmers or organic food, purchasing fruits and vegetables. Increased interest in healthy food may lead, in some cases, to higher orthorexia risk. Indeed, recent studies indicate that symptoms of various eating disorders (e.g., anorexia, bulimia, excessive eating, emotional eating, orthorexia) increased during the lockdown [67,68,69].

The COVID-19 epidemic significantly impacted eating disorder (ED) patients, interfering with the recovery process [97]. Parsons et al. [98] suggested that the pandemic has impacted the experience of ED patients, the experience of service provision and the family situation. Therefore, support is necessary in various forms for people with eating disorders and their families. Rodgers et al. [67] suggested the disruptions to daily routines and constraints to outdoor activities may increase weight and shape concerns and negatively impact eating, exercise, and sleeping patterns, increasing ED risk and symptoms. In contrast, regularizing daily routines, such as healthy diet, sleep, personal hygiene, exercising, leisure/social activities, and practices associated with work or study, can maximize the efficacy to maintain an overall regular daily living and buffer the adverse impact of stress exposure on mental health [99].

Previous research conducted among university students during the COVID-19 pandemic showed the prevalence of sufficient PA level was 62.9% in Switzerland [23], 43% in Ukraine [24], and only 38% in Turkey [5]. These prevalence rates are lower than in similar research performed in university students during the pre-pandemic time [29,30,31,32]. However, university students who improved their dietary habits were also more likely to have healthier lifestyles in other areas, including a higher level of PA [13].

Research indicates the COVID-19 pandemic significantly affected lifestyle, particularly reducing physical activity and changing eating habits [10,11,12,13,14,15,16,17,18,19,20]. Sulejmani et al. [20] found that the weight gained during the lockdown in Kosovo was positively associated with a higher cooking frequency, lower meat and fish consumption, higher fast-food consumption, and no physical activity performance. The study results from Iraqi Kurdistan showed that 12.0% of participants reported improvements in lifestyle, whereas 50.9% declared that their lifestyle deteriorated [16]. These negative changes included the decreased frequency of physical activity, changes in appetite (29.3% felt that their appetite increased while decreased in 14.3%), and weight gain (32.4%). In conclusion, this study is among the earliest studies showing the effect of COVID-19 on eating behavior and lifestyle changes. Furthermore, significant decreases were also observed in the frequency of intake of rice, meat, poultry, fresh vegetables, fresh fruit, soybean products, and dairy products, while significant increases were found in wheat products, other staple foods, and preserved vegetables among Chinese youth [21].

Previous studies indicated that university students demonstrate a relatively low level of healthy behavior, which is related to lack of the parents’ control, lack of enough time, low health literacy, and frequent social gatherings, which is related to excessive substance use and less sleep time [25,26,27,28]. Cena et al. [28] suggested greater emphasis needs to be placed on improving the lifestyle of those university students who will be future healthcare workers.

On the other hand, health literacy was found to be a protective factor from fear of COVID-19 among Vietnamese medical students [63]. A negative association was found between fear of COVID and health-related behaviors, such as smoking and drinking alcohol [63]. Lower FCV-19S scores were presented in male participants, those of older age or in their last academic years, and those able to pay for medication. Fear of COVID-19 is substantially related to wellbeing. Latent profile analysis revealed that 46% of Turkish university students were classified into the sample with high fear of COVID-19 and medium psychological symptoms of stress, anxiety, and depression [65]. Low psychological symptoms and high mindfulness and resilience were found in 38% of participants. A high COVID-19 fear, psychological symptoms, and low mindfulness and resilience were classified among 16% of students. Yalçın et al. [65] evidenced that fear of COVID-19 was positively related to female gender, depression, anxiety, and stress, and negatively to life satisfaction, social support, mindfulness, and resilience. Furthermore, a study conducted among university students from Ecuador showed the mediating role of anxiety in the relationship between fear of COVID and depression [64]. Positive links were also found between fear of COVID-19, the personality trait of neuroticism, social networks use disorder, and smartphone use disorder among Chinese university students [62].

### 4.3. Limitations of the Study

Although this study identified significant evidence of the mediating effect of orthorexia on the association between PA and fear of COVID-19 in a sample of university students, the findings should be interpreted with caution due to the cross-sectional design. Further research should be aimed at performing a longitudinal repeated-measures study for matched samples. Furthermore, the sample represents one technical university from Poland. Therefore, it does not allow us to generalize the results of this study to the population of university students as a whole and the general Polish population. A cross-cultural study could be conducted to compare the present results with other samples from various countries. In addition, self-reported measures included in a survey may also be a source of potential bias. Future research may use observational, experimental, and psychophysiological methods to assess PA, orthorexia, and fear of COVID-19. Several problems as a potential source of bias are related to the online survey method, including self-selection bias, representation bias, and unknown response rates. Therefore, these studies should be repeated on a representative sample of university students in paper and pencil form. Moreover, alternative measures can be used to assess PA, orthorexia, and COVID-19 anxiety, which may increase the reliability of the research in the future. Finally, mental health was not analyzed in this study, so it is unclear to what extent the fear of COVID-19 was associated with depression, generalized anxiety disorder (GAD), specific phobias, obsessive-compulsive disorder (OCD), or post-traumatic stress disorder (PTSD). Future studies using the fear of COVID-19 scale should control participants’ diagnosis and risk of mental disorders.

## 5. Conclusions

The study found a mechanism by which healthy lifestyle behaviors affect the fear of COVID-19 during the lockdown. These findings confirmed the indirect effects of orthorexia on the relationship between exercise and fear of COVID-19. As expected, healthy behavior related to PA and diet can decrease fear of COVID-19. However, physical activity was found here as a complex variable, and its various aspects may also have opposite effects on the fear of COVID-19. Although high PA decreases fear of COVID, exercise addiction and orthorexia tendencies can trigger an opposite process that limits its beneficial impact on the dependent variable. The addictive, obsessive, and compulsive behaviors associated with a healthy lifestyle can increase COVID-19 anxiety and decrease wellbeing. It is also possible that there is a reciprocal association between fear of COVID-19 and addictive behavior related to health. Rodgers et al. [67] suggested that fear of COVID-19 may increase ED symptoms, specifically related to health concerns due to the pandemic and social isolation, or by the pursuit of restrictive diets focused on increasing immunity. In addition, the disruptions to daily routines and constraints to outdoor activities may increase weight and shape concerns and negatively impact eating, exercise, and sleeping patterns, increasing ED risk and symptoms, including orthorexic restrictive eating. Evaluating and assessing all factors contributing to EDs across different cultural settings is essential to better understand the impact of the pandemic on ED risk and recovery. In conclusion, individuals prone to addictive behavior should increase their efforts to maintain their daily routine regardless of changing circumstances during the lockdown and following waves of the COVID-19 pandemic. In addition, some preventive or intervention strategies can help maintain wellbeing.

Cognitive restructuring (as coping strategy) showed an inverse association with the students’ fear of COVID-19 [60]. Therefore, clinical interventions could train effective coping strategies for daily stress to improve students’ wellbeing. Developing programs for promoting healthy lifestyle behaviors among students is also recommended [100]. University institutions should adopt support mechanisms to alleviate psychological impacts on students during the COVID-19 pandemic. Changes in eating and exercise behaviors need to be acknowledged and monitored during the COVID-19 pandemic for potential long-term consequences [19]. It is crucial to detect orthorexia symptoms and high fear of COVID-19 levels early during the pandemic to modulate daily behaviors to prevent long-term detrimental consequences [69].

## Figures and Tables

**Table 1:** Demographic characteristics of the sample.

Demographic Variable	*n*	%
Gender		
Women	222	47%
Men	251	53%
Relationship status		
In a relationship	281	59%
Single	192	41%
Place of residence		
Village	232	49%
Town	172	36%
City	61	13%
Agglomeration	8	2%
Department		
Civil Engineering and Architecture	61	13%
Economics and Management	104	22%
Electrical Engineering, Automatic Control, and Informatics	96	20%
Production Engineering and Logistics	17	4%
Mechanical	23	5%
Physical Education and Physiotherapy	172	36%
Study level		
First degree (Bachelor’s, three-years)	322	68%
Second degree (Master’s, two-years)	74	16%
Uniform Master’s study (five-years)	74	16%
Doctoral	3	1%
Year of study		
1	162	34%
2	158	33%
3	115	24%
4	29	6%
5	9	2%
Type of study		
Full-time	428	90%
Part-time	45	10%

**Table 2:** Descriptive statistics, normality tests, and reliability coefficients of measured variables.

Variable	*M*	*SD*	*Mdn*	*Min*	*Max*	Skewness	Kurtosis	*W*	*p*
Fear of COVID-19	12.93	5.67	11.00	7.00	35.00	1.36	1.98	0.87	<0.001
TON-17 total score	45.84	9.66	46.00	17.00	85.00	−0.04	1.17	0.99	<0.001
Control of food quality	16.34	4.57	17.00	6.00	30.00	−0.01	−0.07	0.99	<0.001
Fixation on health and healthy diet	17.87	3.89	18.00	5.00	25.00	−0.68	0.52	0.96	<0.001
Disorder symptoms	11.62	4.67	11.00	6.00	30.00	1.23	1.96	0.90	<0.001

**Table 3:** Differences in orthorexia and fear of COVID between female and male participants and active and inactive people.

	Men		Women				Active		Inactive			
	(*n* = 251)		(*n* = 222)				(*n* = 222)		(*n* = 251)			
Variables	*M*	*SD*	*M*	*SD*	*t* (471)	*d*	*M*	*SD*	*M*	*SD*	*t* (471)	*d*
FCV-19S	12.29	5.82	13.65	5.41	2.63 **	0.24	12.03	5.11	13.73	6.01	3.29 **	0.30
TON-17	45.88	10.35	45.79	8.84	−0.10	−0.01	47.02	8.88	44.78	10.21	−2.53 *	−0.23
CFQ	16.28	4.71	16.41	4.42	0.32	0.03	17.01	4.55	15.75	4.52	−3.01 **	−0.28
FHHL	17.53	4.05	18.26	3.66	2.06 *	0.19	18.70	3.50	17.14	4.07	−4.46 ***	−0.41
DS	12.07	5.29	11.11	3.80	−2.24 *	−0.21	11.31	4.40	11.90	4.89	1.36	0.13

Note. FCV-19S = Fear of COVID-19 Scale; TON-17 = 17-item Test of Orthorexia Nervosa; CFQ = Control of Food Quality; FHHL = Fixation on Health and Healthy Lifestyle; DS = Disorder Symptoms. * *p* < 0.05, ** *p* < 0.01, *** *p* < 0.001.

## Data Availability

The data presented in this study are openly available in Mendeley Data at doi: 10.17632/vz7wktbvkd.1.
